# Therapeutic potential of mesenchymal stem/stromal cells (MSCs)-based cell therapy for inflammatory bowel diseases (IBD) therapy

**DOI:** 10.1186/s40001-023-01008-7

**Published:** 2023-01-27

**Authors:** Mohamed J. Saadh, Maria V. Mikhailova, Soheil Rasoolzadegan, Mojgan Falaki, Roozbeh Akhavanfar, José Luis Arias Gonzáles, Amir Rigi, Bahman Abedi Kiasari

**Affiliations:** 1 Department of Basic Sciences, Faculty of Pharmacy, Middle East University, Amman, 11831 Jordan; 2 I.M. Sechenov First Moscow State Medical University (Sechenov University), Moscow, Russia; 3 Department of Surgery, School of Medicine, Shahid Beheshti University of Medical Sciences, Tehran, Iran; 4 Department of Internal Medicine, Shahid Beheshti University of Medical Sciences, Tehran, Iran; 5 School of Medicine, Isfahan University of Medical Sciences, Isfahan, Iran; 6 Pontificia Universidad Católica del Perú, Lima, Peru; 7 Department of Nursing, Young Researchers and Elite Club, Zahedan Branch, Azad University, Zahedan, Iran; 8 Virology Department, Faculty of Veterinary Medicine, The University of Tehran, Tehran, Iran

**Keywords:** Mesenchymal stem/stromal cells (MSCs), Inflammatory bowel disease (IBD), Inflammation, Intestinal integrity, Cytokines

## Abstract

Recently, mesenchymal stem/stromal cells (MSCs) therapy has become an emerging therapeutic modality for the treatment of inflammatory bowel disease (IBD), given their immunoregulatory and pro-survival attributes. MSCs alleviate dysregulated inflammatory responses through the secretion of a myriad of anti-inflammatory mediators, such as interleukin 10 (IL-10), transforming growth factor-β (TGFβ), prostaglandin E2 (PGE2), tumor necrosis factor-stimulated gene-6 (TSG-6), etc. Indeed, MSC treatment of IBD is largely carried out through local microcirculation construction, colonization and repair, and immunomodulation, thus alleviating diseases severity. The clinical therapeutic efficacy relies on to the marked secretion of various secretory molecules from viable MSCs via paracrine mechanisms that are required for gut immuno-microbiota regulation and the proliferation and differentiation of surrounding cells like intestinal epithelial cells (IECs) and intestinal stem cells (ISCs). For example, MSCs can induce IECs proliferation and upregulate the expression of tight junction (TJs)-associated protein, ensuring intestinal barrier integrity. Concerning the encouraging results derived from animal studies, various clinical trials are conducted or ongoing to address the safety and efficacy of MSCs administration in IBD patients. Although the safety and short-term efficacy of MSCs administration have been evinced, the long-term efficacy of MSCs transplantation has not yet been verified. Herein, we have emphasized the illumination of the therapeutic capacity of MSCs therapy, including naïve MSCs, preconditioned MSCs, and also MSCs-derived exosomes, to alleviate IBD severity in experimental models. Also, a brief overview of published clinical trials in IBD patients has been delivered.

## Introduction

**Fig. 1:**
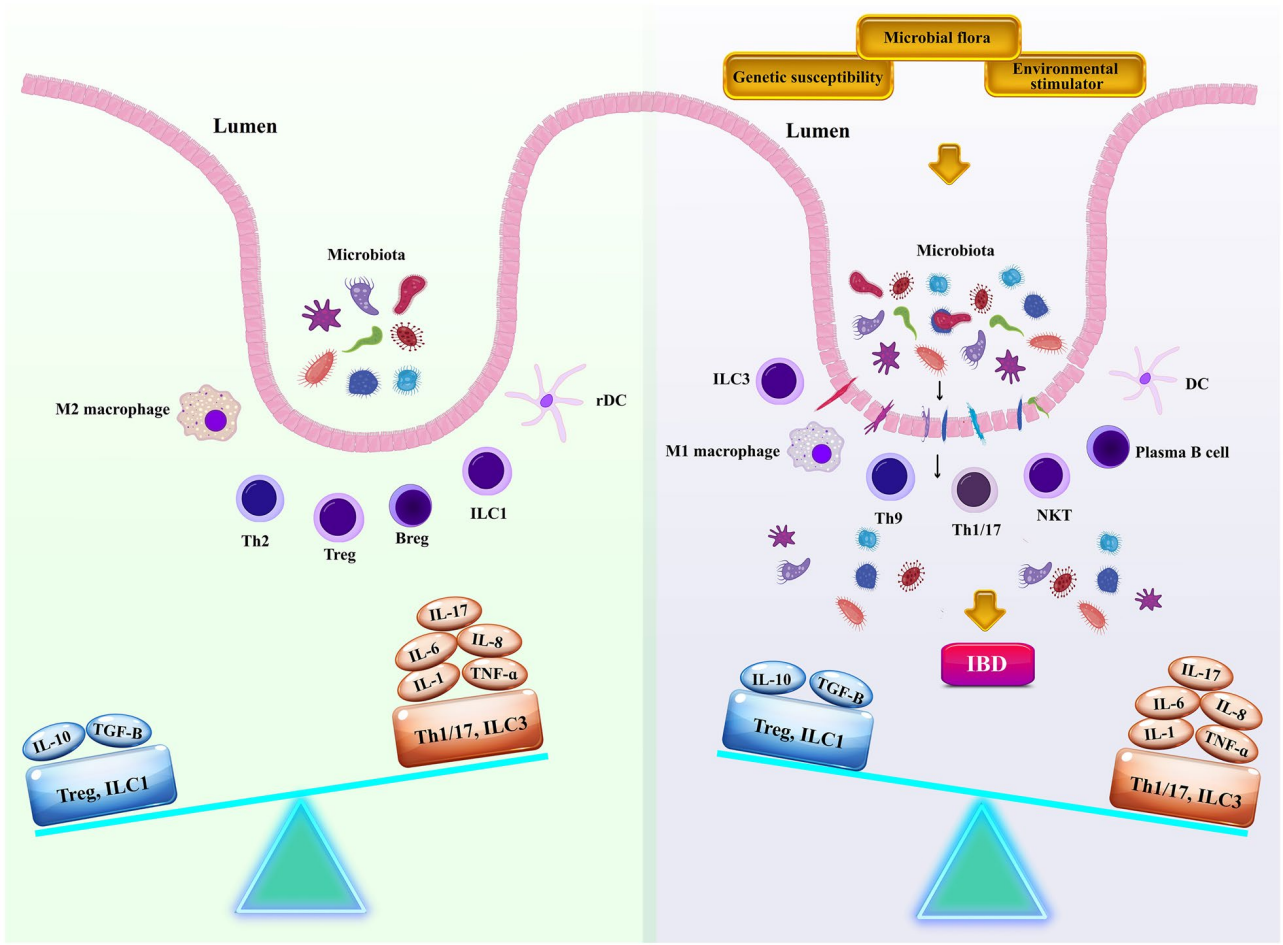
The IBD pathogenesis. The interactions between environmental factors, genetic susceptibility, and microbial flora may perturb intestinal hemostasis and thus induce the transduction of dysregulated immune responses and underlie resultant tissue damage. Inflammatory bowel diseases (IBD), innate lymphoid cells (ILC), T helper cell (Th), interleukin (IL), transforming growth factor-beta (TGF-β), regulatory B cells (Bregs), regulatory T cells (Tregs), regulatory dendritic cells (rDCs), natural killer T (NKT) cells, tumor necrosis factor α (TNFα)

Inflammatory bowel disease (IBD) is a chronic disease of unknown origin characterized by serious inflammation and mucosal destruction in the intestine [1]. There are two chief forms for IBD: Crohn’s disease (CD), which is most communal in the colon and terminal ileum and can target the entire gastrointestinal (GI) tract, and ulcerative colitis (UC), which is a mucosal inflammation comprising the rectum and colon [2]. Nowadays, IBD is typically suggested to be induced through interfaces among environmental [3], genetic [4], infectious [3, 5], and immune factors [6]. Indeed, IBD is initiated and preceded by the coinciding of several genetic and environmental stimuli, which finally disturb the immune–microbiome axis (Fig. 1). Now, shedding light on the various aspects of IBD pathogenesis, with a particular emphasis on immunological pathogenesis to introduce novel and effective therapeutic approaches is of paramount importance.

The existence of large quantities of symbionts near the epithelial surface of the intestine brings about a huge challenge to the host since it must constrain the triggering of dysregulated inflammatory responses to the microorganisms, while supporting its aptitude to trigger strong immune responses for attacking pathogens [6, 7]. The results derived from the genome-wide association studies (GWAS) have evidenced the correlation between the innate and the adaptive immune system in adjusting the sensitive equilibrium of the mucosal immune system [8, 9]. However, accumulating proof implies that a dysregulated immune response against the microorganisms in the intestine may lead eventually to IBD onset and progress in genetically susceptible individuals [10]. T helper cell (Th) type 1 (Th1) and Th17-mediated immune responses with improved levels of interleukin (IL)-1β, tumor necrosis factor-alpha (TNF- α), interferon-gamma (IFN-γ), IL-6, IL-8, IL-12, IL-17, and IL-23 are typically observed in CD patients, while Th2-associated immunological response with enhanced IL-4, IL-5, and IL-13 levels are a common pathological sign in UC patients [10–12]. Given the central role of immune response in IBD pathogenesis, scientists are trying to recognize and advance new and more efficient treatments based on the alleviation of dysregulated immune responses in IBD patients, leading to reduced disease severity and improved life qualities.

Growing reports show that mesenchymal stem/stromal cells (MSCs) trigger robust anti-inflammatory and immunoregulatory effects, causing improved tissue repair [13, 14]. MSCs have displayed appreciated therapeutic benefits in a large number of IBD animal studies and some clinical trials on patients suffering from IBD [15, 16]. Recent studies showed that CD-derived diseased mesentery tissue MSCs was found to lose their immunosuppressive capability in the treatment of CD by distinct regulation of pathogenic T-cell responses and/or T-cell infiltration into the colon [17]. However, exogenous MSCs could be an ideal source for IBD therapy. MSCs can engraft the intestinal mucosa, regulate inflammation and thus repair injured tissues. Transplanted MSCs, in fact, restore gut microbiome alteration and promote efficient eradication of pathogenic bacteria. MSCs-mediated immunomodulation is exerted through secretion of the multiple anti-inflammatory mediators, such as TNF-stimulated gene 6 (TSG-6), prostaglandin E2 (PGE2), indoleamine 2,3-dioxygenase (IDO), and transforming growth factor-beta (TGF-β) [18, 19]. MSCs prohibit the Th1/17-mediated inflammatory responses, thus attenuating the levels of the inflammatory molecules and offering a suitable milieu for tissue recovery [20, 21]. They also potentiate the release of the inhibitory factor IL-10 by targeting macrophage polarization [22]. Apart from their capability to moderate immune response, MSCs augment intestinal epithelial cell (IECs) survival and proliferation and simultaneously upregulate the expression of tight junction (TJs)-related molecules in such IECs [23, 24]. These effects, in turn, maintain the intestinal integrity and support barrier function [25]. Noteworthy, current studies have shown that using the pre-conditioned MSCs [26, 27] or MSCs-derived exosome [28, 29], as an innovative cell-free approach, can be more favored strategy compared to naïve MSCs cells therapy. Modified MSCs or MSCs-derived exosomes could circumvent MSCs' low migration and engraftment to colon tissue, making them a rational and effective therapeutic strategy [30]. This review provides an overview for elucidating the therapeutic effects of MSCs therapy in IBD, with a special focus on recent in vivo studies.

## Immunological pathogenesis of IBD

The IBD is an autoimmune condition of the GI tract characterized by loss of tolerance to self-antigens and intestinal flora, preceded by over-activated and destructive immune responses. Although the detailed etiopathogenesis of IBD has remained elusive, genetic predisposition, dysbiosis, environmental factors, and aberrant immune responses have all been implicated [31]. The main stimuli of persistent inflammation and tissue destruction in IBD patients are excessive immune cell infiltration in colonic lesions and their products, which upregulate pro-inflammatory cytokines and chemokines expression levels [32].

Several immunopathological studies on IBD have demonstrated that IL-17-producing cells, Th17, play a central role in IBD development, while its suppression in patients with acute colitis may reduce inflammation and thus mitigate disease severity [33, 34]. The IL-17 and signal transducer and activator of transcription 3 (STAT3) are upregulated in inflamed colon tissue as compared to healthy counterparts [35]. Mechanistically, IL-17 activates STAT3, which provokes chronic inflammatory immune responses; hence IL-17 inhibition mediated by phosphorylated STAT3 can potentially reverse inflammation and IBD progression [36]. Besides, the bidirectional interactions of Tregs and Th17 modulate Th17-mediated immune responses. In fact, Th17 and Tregs interactions and a balance between them is a crucial prognostic indicator in the immunopathogenesis of IBD [37]. In light of this, experimental colitis models have indicated that Foxp3^+^ Tregs have anti-inflammatory functions in the intestine by suppressing Th17 responses and thus reducing Th17/Treg ratio [38, 39].

Other cytokines with significant implications in IBD pathogenesis are the IL-1 family members. IL-1β expression and function are mostly associated with innate immune cells, such as macrophages and monocytes. However, in UC patients, it is expressed in colonic mucosa and enhances inflammation [40]. Similarly, another IL-1 family member, IL-18, is abundantly expressed in the mucosa of CD patients, enhancing Th1 response while interfering with immunoregulatory cytokine release from mucosal T cells, such as IL-10 [41]. Despite contradictory findings on the IL-10 level in IBD patients, it is well documented that IL-10 down-regulation is associated with detrimental effects and disease progression [42]. Moreover, IL-33 and its cognate receptor, ST2, are upregulated in UC patients and can positively regulate IL-5 and IL-13 expression, leading to enhanced Th-2 response and tissue protection [43, 44]. Similarly, TNF-α could enhance the levels of IL-1β, IL-6, and IL-33 in IBD patients, thus its level negatively correlates with the clinical outcome of these patients [45, 46]. Also, TGF-β could dampen inflammation by suppressing IL-33, promote epithelial compensation and fibrosis and maintain intestinal homeostasis and mucosal tolerance [47]. Furthermore, IL-6 and its soluble receptor is overexpressed in UC and CD patients and promotes inflammation via STAT3 activation and substantially contributes to the development of colorectal cancer (CRC) in UC patients [48, 49].

A growing body of evidence indicates that chemokines are critical not only for systemic inflammation, but also for homeostasis and immune regulation [50, 51]. Multiple chemokines are released by a variety of immune cells infiltrated in IBD lesions, including macrophages and neutrophils. These cells serve key roles in the development and progression of IBD [52]. In this context, upregulated levels of chemokine ligand (CCL) 2, CCL4, CCL7, and C-X-C motif chemokine ligand 10 (CXCL10) in IBD tissues outlines their significance in immune infiltration and disease severity [53]. These discoveries have paved the path for the development of therapeutic strategies to target various chemokines in IBD patients.

## Interaction between MSCs and immune cells

Given their unique and robust immunoregulatory competencies, MSCs moderate immune responses during tissue repair and offer a suitable milieu for tissue regeneration [54]. Based on the molecular and cellular analysis, a diversity of soluble factors in association with cell contact-mediated process in response to the immune cells or other stimuli’s involves in MSCs-induced immunomodulation [55, 56]. MSCs principally regulate the adaptive and innate immune responses through the influencing T cells activation and differentiation and dendritic cells (DCs) maturation. For instance, MSCs inhibit DCs type 1 (DC1) activation to downregulate TNF-α secretion while promoting IL-10-secreting DC2 performance in rodent models of IBD [57]. MSCs also affect DCs to substantially produce galectin 3 (Gal-3) in rodents with colitis [58]. The Gal-3 is a lectin that participates in immunosuppression and adjust various functions such as cellular homeostasis [59].

Also, they downregulate B-cell as well as NK cells activation and conversely promote the growth of T regulatory (Treg) cells by both secretions of soluble factors and cell-to-cell contact [60, 61]. MSCs also inhibit the activation of pro-inflammatory macrophages (M1Mφ), TH1, and TH17, while supporting TH2 and anti-inflammatory macrophages (M2Mφ) largely by secreting IL-10 and TGF-β [62].

**Fig. 2:**
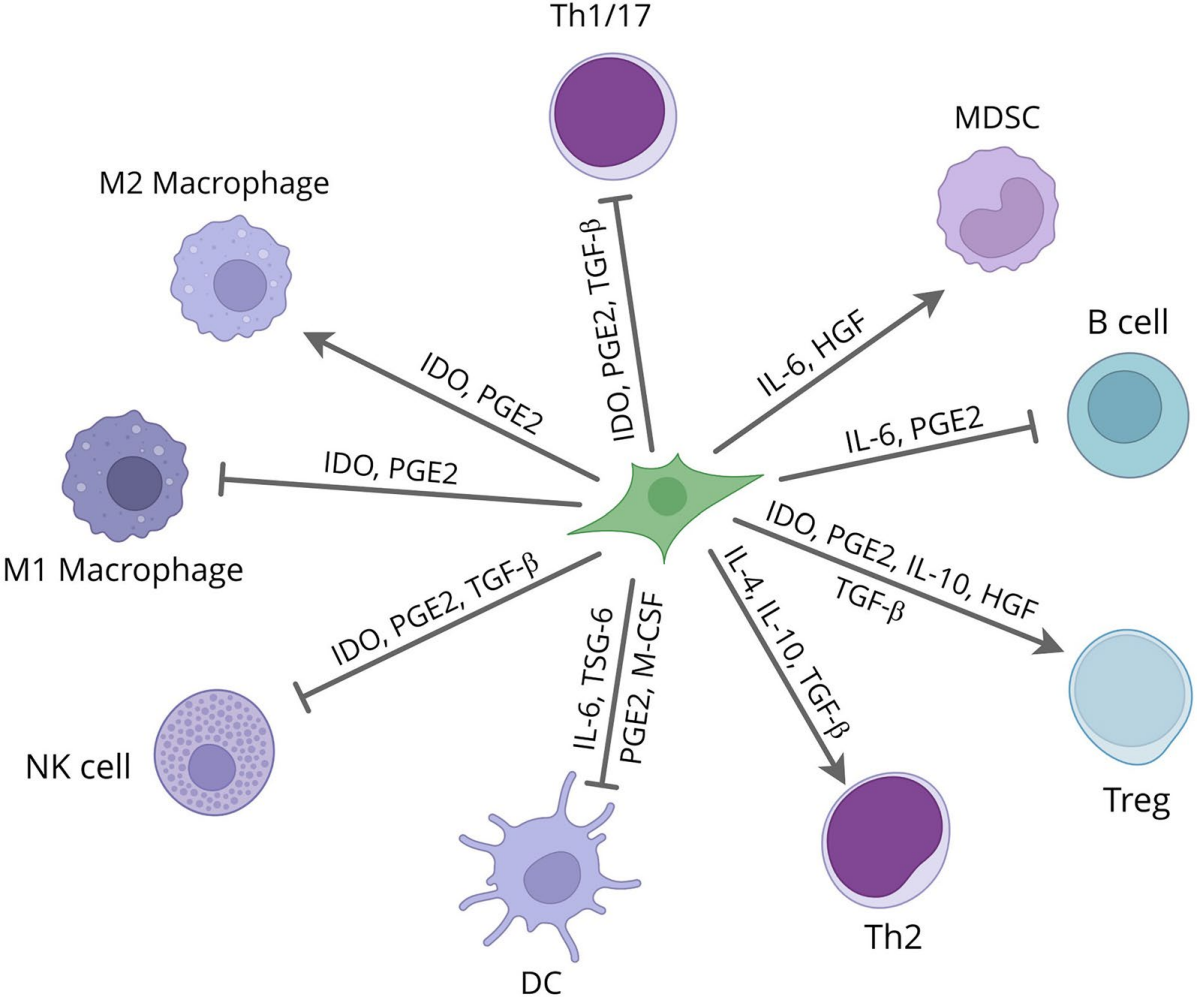
Underlying mechanisms behind the immunomodulatory attributes of MSCs

The inhibitory effects of MSCs on immune cells are depicted in Fig. 2.

The effects of the MSCs on immune cells functions may vary depending on the MSCs sources [63] and also target tissue [64]. For instance, immunoregulatory potential of adipose tissue (AT)-derived MSCs is stronger than bone marrow (BM)-derived MSCs on peripheral blood mononuclear cell (PBMC) proliferation. [65]. Also, in terms of production of IL-2, IL-4, IL-13, and GM-CSF, chorionic plate-derived MSCs (CP-MSCs) have superiority over BM-MSCs and AT-MSCs [66]. Another study also signified that the inhibitory effects of the BM-MSCs are higher than placenta‐derived MSCs (PD‐MSCs) on T cell activation [67]. In sum, there is universal agreement concerning the immunomodulatory effects of MSCs; however, further study to elucidate the detailed biological mechanisms behind these effects is required.

## The rationality of MSCs therapy in IBD

### Anti-inflammatory effects

The CD is usually described as a Th1/17-associated disorder since the main inflammatory cytokines in this condition are the Th1/17-related molecules like IL-12, IL-17, IFN-γ, and TNF-α. On the other hand, UC is typically described as a Th2-associated disease due to the upregulated intestinal expression of the Th2-related cytokines (e.g., IL-5, IL-13, and IL-4) [68, 69]. In light of this, the immunological basis of the IBD implies that MSCs-based cell therapies can be a rational therapeutic modality to alleviate IBD pathological signs due to their capacity to moderate inflammatory responses.

Accumulating evidence indicates that MSCs modify immunological response through various mechanisms, such as suppression of T cell-mediated inflammatory responses, targeting dendritic cells (DCs) maturation and phenotype shift and also promoting anti-inflammatory M2 macrophages polarization [70]. In light of this, MSCs administration (1 × 10^6^ cells) stimulated DCs differentiation into regulatory DCs (rDCs) with potent immunoregulatory properties and enhanced colon length, body weight, and overall survival (OS) rate in DSS-induced colitis mice [71]. The rDCs are also implicated in inducing and maintaining the homeostasis of Tregs. MSCs transplantation also decreased expression of IL-6, TNF-α, and IFN-γ and concurrently increased the levels of IL-10, TGF-β, and forkhead box protein P3 (Foxp3), a crucial regulator of Tregs, in colon tissues [71]. Also, MSCs could decrease CD4 + T cell proliferation, inhibit Th1/Th17 cells activation, while inducing Th2 cells function [72]. These events finally diminish pro-inflammatory IL-17 and IFN-γ, and improve TGF-β, IL-10 levels in colon tissue of MSCs-treated IBD models [73]. As well, MSCs could improve the Tregs population and decrease IFN-γ secretion by natural killer (NK) cells in inflamed tissue [74]. Regardless of the pro-inflammatory cytokines, systemic administration of MSCs could affect T-box expressed in T cells (T-bet) and retinoid-related orphan receptor gamma(t) (RORγt) expression in T cells [21]. T-bet and RORγt, as a central regulator of Th1 and Th17 cells, respectively, act as causative factors in IBD progress. Thus, their inhibition by MSCs-secreted mediators could lead to the suppression of Th1/Th17-related pathological events in IBD patients.

Irrespective of cited mediators, PGE2, hepatocyte growth factor (HGF), IDO, and also nitric oxide (NO) involve in MSCs-mediated immunoregulation [14, 75–77]. Park and coworkers (2018) exhibited that AT-derived MSCs administration could alleviate inflammatory response in DSS-induced mice with chronic colitis by promoting M2 macrophage polarization as a result of PGE2 secretion [78]. In vitro, AT-MSCs-secreted PGE2 attenuated the proliferation of THP-1 cells, a human leukemia monocytic cell line, and diminished the secretion of inflammatory cytokines IL-1β and IL-18 by macrophages [78]. AT-MSC-secreted PGE2 also attenuated inflammation by enhancing Foxp3 + Tregs numbers in DSS-induced mice colitis [79]. In the DSS-induced colitis model, recent results also exhibited that (umbilical cord blood) UCB-derived MSCs pre-conditioned with IL-1β and IFN-γ could diminish Th1 cell differentiation and stimulate Tregs differentiation in colon tissue, which mainly caused by PGE2 and IDO delivery to target tissue [80]. Also, there is clear evidence that HGF and TSG-6 released by MSCs could attenuate colitis [81]. Li et al. [82] revealed that dental pulp stem cells overexpressing HGF (HGF-DPSCs) decreased intestinal mucosa damage in part through the transdifferentiating into an intestinal stem cell (ISC)-like cells, stimulating ISC-like cell growth, inhibition of the intense immunological responses, and plummeting oxidative stress in DSS-induced ulcerative colitis. Besides, TSG6, a 30-kDa protein, which is secreted by activated macrophages and MSCs, inhibits the association of toll-like receptor 4 (TLR4) with myeloid differentiation primary response 88 (Myd88), thereby down-regulating nuclear factor (NF)-κB activation [83, 84]. In addition, TSG6 stimulates a macrophage phenotypic shift from M1 to M2, having important role in alleviating DSS-induced colitis [85], whereas TSG6^−/−^ MSCs did not suppress the mucosal inflammatory response in colitis' mice [86]. Recently, bioinformatics analyses showed that UC includes communication between macrophages and enterocytes via ligand–receptor pairs, and AT-MSCs may alleviate this condition by communicating with macrophages to block inflammation [87]. Besides, Liu et al. [88] declared that MSCs could improve DSS-induced colitis in part by adjusting the Tregs–immunoglobulin A (IgA) response, increasing the secretion of IgA, and enabling the restoration of intestinal microbiota. These findings offer a potent therapeutic mechanism for MSCs in the IBD treatment.

### Supporting intestinal barrier

The intestinal barrier makes a separation between the body and the contents of the intestine. It consists of various sections, including a mucus layer comprising antibacterial peptides lining the luminal surface of the epithelium, the epithelial cell monolayer, junctional proteins, intraepithelial lymphocytes (IELs), and also a subepithelial layer of extracellular matrix (ECM) and mesenchymal cells like myofibroblasts and fibroblasts [89, 90]. The integrity of the intestinal barrier depends on several contributors, such as strong innate immune responses, epithelial paracellular permeability, epithelial cell integrity, and the secretion of mucus [91]. The intestinal barrier provides a shield versus potentially damaging molecules and also pathogenic bacteria, thus supporting intestine immune homeostasis [92]. Intestinal epithelial cells (IECs) play a critical role in maintaining the barrier's integrity given their anatomical and functional location. On the luminal side, IECs release and adjust the contents of the mucus layer, whereas they interrelate and cross-talk with the underlying cells on the basolateral side [93, 94]. In health, IECs underlie a persistent intestine barrier due to the action of tight junction (TJ) proteins [95]. The TJ typically is formed by transmembrane proteins like claudins and occludin accompanied by cytoplasmic proteins like zonula occludens (ZO)-1 [96]. In normal conditions, the expression of TJ proteins contributes to the adjusting of colonic permeability during a regulated process in the intestine [97]. Notwithstanding, these proteins are susceptible to colitis and reduced expression and function of epithelial occludin have been found in IECs of UC patients [98, 99]. Indeed, impairment in the function of intestinal epithelial barrier is a prominent property of IBD.

Recent reports have exhibited that BM-MSCs transplantation may promote the proliferation and inhibit the apoptosis of IECs, thus favoring cellular homeostasis and intestinal integrity in vivo [100]. Also, MSC-derived conditioned media (CM) was capable of promoting injured fetal intestinal epithelial cells (FIEs) proliferation in vitro potently via IL-6, HGF, and vascular endothelial growth factor (VEGF) delivery, largely contributing to IECs integrity and simultaneously suppressing apoptotic pathways [101]. As well, activation of phosphatidylinositol-3-kinase (PI3K)/AKT pathways in IECs and resultant IECs proliferation were observed upon MSCs-derived CM injection by intravenous route in DSS and TNBS-induced colitis [102]. PI3K/Akt pathway controls IECs proliferation via modification of the availability of functional cyclin D1 protein [103]. In addition, Yang et al. [18] revealed that induced pluripotent stem cells (iPSC)-derived MSCs could boost IECs proliferation to ameliorate mucosal healing in a mice colitis model by TSG-6 secretion. In vitro, TSG-6 could promote Akt phosphorylation in mice colonoids (primary cultures derived from intestinal crypts), reflecting the key role of Akt activation in the TSG-6-mediated proliferation of IECs [18]. Similarly, administration of iPSC-MSCs promoted IECs proliferation, raised the Lgr5 + ISCs frequencies, and potentiated intestinal angiogenesis in colitic rodents [16]. Positive regulation of Lgr5 + ISCs proliferation and differentiation, as shown by exogenous PGE2 injection, may augment intestinal integrity and promote mucosal healing in IBD patients [104].

In addition to targeting IECs and ISCs biological process, treatment of colitic rats with AT-MSCs-derived CM enhanced the expression of mucin 2 (Muc2), the major colonic mucin which its deficiency leads to the perturbation of colon tissue hemostasis [105]. Muc2^−/−^ mice have bacteria in direct communication with the IECs and far down in the crypts, sustaining inflammation and cancer [106]. AT-MSCs-derived CM also upregulated the expression of TJ-related proteins, claudin-1, and ZO-1 [105]. Both claudin-1 and ZO-1 are known for their barrier-forming abilities and serve urgent roles in protecting intestinal function and integrity [107]. Recent results have shown that miR-181 containing MSC-derived exosome [108] and also superoxide dismutase 3 (SOD3) overexpressing MSCs [109] can promote claudin-1 and ZO-1 expression and consequently upgrade intestinal barrier function, conferring the unique competencies of MSCs-based cell therapies to support intestinal barrier in IBD patients.

## Preclinical studies based on MSCs therapy in IBD

### Naive MSCs

A growing body of proof suggests MSCs as a promising therapeutic tool for IBD treatment mainly due to their immunomodulatory and anti-inflammatory attributes. In addition, they stimulate colon epithelial integrity and repair by increasing the proliferation of IECs in part by enhancing circulating insulin-like growth factor-1 (IGF-1) in colitis rodents [110]. In this section, we have focused on the transplantation of naïve MSCs in IBD preclinical models to elucidate the affiliated mechanism behind the observed in vivo desired effects (Table 1).

**Table 1:** Naive MSCs transplantation in IBD animal models

Diseases	Source	Model	Results	Refs.
TNBS-induced colitis	UC	BALB/c mice	Reducing colitis by suppression of the Th1/17 activation	[227]
DSS-induced colitis	BM	C57BL/6 mice	Reducing the numbers of neutrophils in colon tissue and metalloproteinase activity in the mucosa while promoting the Tregs population	[85]
DSS-induced colitis	BM	BALB/c mice	Attenuation of TNF-α and IL-1β levels in both the inflamed colon and serum	[68]
DSS and TNBS-induced colitis	UC	BALB/c mice	Amelioration of colitis by up-regulating NOD2/COX2 signaling pathways	[69]
TNBS-induced colitis	Placenta	BALB/c mice	Promoting the therapeutic potential of MSCs by improving the PGE2-mediated M2 macrophage polarization by combination therapy with chitosan-based hydrogel	[228]
TNBS-induced colitis	AT BM	Wistar rats	Promoting VEGF and TGF-β levels while attenuating TNF-α and IL-1β	[229]
TNBS-induced colitis	BM	Wistar rats	MSCs’ partial differentiation into IECs and secreting VEGF and TGF-β1	[230]
DSS-induced colitis	BM	C57BL/6 mice	Recruiting M2 macrophage to reduce colitis severity by secretion of the TGFβ1	[231]
AOM and DSS-induced colitis	UC	C57BL/6 mice	Enhancing Treg cells differentiation by MSCs-secreted TGF-β	[232]
DSS-induced colitis	AT	C57BL/6 mice	Stimulation of the polarization of the M2 macrophages by MScs-secreted TSG-6	[233]
DSS-induced colitis	BM	C57BL/6 mice	Suppression of dendritic cells (DCs) activation by the release of galectin 3	[58]
AOM and DSS-induced colitis	BM	C57BL/6 mice	Amelioration of histological damage along with reducing TNF-α, IL-1β, and IL-6 levels and down-regulation of STAT3 phosphorylation in colon tissue	[234]
DSS-induced colitis	UC	C57BL/6 mice	Eliciting anti-inflammatory response in colon tissue by improving Treg/Th17 cells ratio in the spleen and mesenteric lymph nodes	[235]
DSS-induced colitis	BM	C57BL/6 mice	Improved levels of IDO and IL-10 in serum	[236]
DSS-induced colitis	BM	C57BL/6 mice	Improving IL-10 levels, the inflamed colons	[237]
DSS-induced colitis	BM	BALB/c mice	Potentiating intestinal integrity by up-regulation of E-cadherin expression	[25]
DSS-induced colitis	UCB	C57BL/6 mice	Reducing T cell infiltration into the inflamed colon while promoting Tregs frequency	[114]
DSS-induced colitis	UC	C57BL/6 mice	Attenuation of disease activity index (DAI), histological colitis scores along with promoting body weight	[22]
DSS-induced colitis	BM	C57BL/6 mice	Induction of DCs differentiation into rDCs, leading to ameliorated chronic colitis	[71]
TNBS-induced colitis	BM	Hartley guinea pigs	Diminishment of the immune cell infiltration to damaged colons and also reducing neuronal loss in colon tissue	[238]
TNBS-induced colitis	AT BM	Hartley guinea pigs	Amelioration of enteric neuropathy and plexitis	[239]
DSS-induced colitis	BM	C57BL/6 mice	Improvement of the MSCs therapeutic effects by human cord blood-derived platelet lysate	[126]
DSS-induced colitis	UC	C57BL/6 mice	Amelioration of colon and liver pathology by attenuating the LPS levels and TNF-α, IFN-γ, IL-1β, IL-17A, TLR4, TRAF6, and NF-κB expression in inflamed colon	[240]
TNBS-induced colitis	AT	C57BL/6 mice	Reducing colitis severity and diarrhea and enhancing body weight and survival	[241]
DSS-induced colitis	AM	SD rats	Reducing DAI score, weight loss, colon shortening, and the histological colitis score in association with down-regulation of TNF-α, IL-1β, and MIP	[242]
TNBS-induced colitis	AT	Hartley guinea pigs	Attenuation of weight loss, colonic tissue damage, and immune cell infiltration	[243]
DSS-induced colitis	AT	C57BL/6 mice	Enhancing Tregs by MSCs-secreted PGE2	[79]
TNBS-induced colitis	BM	SD rats	Enhancing MSCs recruitment to the colonic mucosa by combination therapy with G-CSF leads to reduced DAI, MPO activity	[125]
TNBS-induced colitis	UC	BALB/c mice	Improvement of CD5 + B cells and Tregs while decreasing Th1 cells, Th17 cells, and various pro-inflammatory cytokines levels in inflamed colon	[116]
DSS-induced colitis	BM	C57BL/6 mice	Improvement of IL-10 secretion by MSCs upon combination therapy with wogonin	[128]
TNBS-induced colitis	BM	C57BL/6 mice	Exerting gastrointestinal mucosal tissues repair by MSCs-secreted IGFBP7	[244]

*MSCs* mesenchymal stem/stromal cells, *BM* bone marrow, *UC* umbilical cord, *UCB* umbilical cord blood, *AT* adipose tissue, *TNBS* 2,4,6-trinitrobenzene sulfonic acid, *DSS* dextran sulfate sodium, *AOM* azoxymethane, *Th* T helper 1/2/17, *FOXP3* Forkhead box P3, *IL-1* interleukin-1β, *TNF-α* Tumor necrosis factor-alpha, *NOD2* nucleotide-binding oligomerization domain-containing protein 2, *COX-2* cyclooxygenase-2, *TGF-β* transforming growth factor-beta, *PGE2* prostaglandin E2, *VEGF* vascular endothelial growth factor, *Tregs* regulatory T cells, *TSG-6* TNF-stimulated gene 6, *STAT3* signal transducer and activator of transcription 3, *IDO* indoleamine 2,3-dioxygenase, *IFN-γ* interferon-gamma, *IL* interleukin, *rDCs* regulatory dendritic cells, *TLRs* toll-like receptors, *TRAF6* tumor necrosis factor receptor-associated factor 6, *NF-κB* nuclear factor kappa B, *NLRP3* NLR family pyrin domain containing 3, *G-CSF* granulocyte colony-stimulating factor, *MPO* myeloperoxidase, *IGFBP7* insulin-like growth factor-binding protein 7, *MIF* migration inhibitory factor

Recent studies display that intraperitoneally injected MSCs (2 × 10^6^) could diminish colitis development and also decrease serum levels of IL-1β, IL-12, and IL-6 in vivo [58]. The anti-inflammatory effects might arise from the inhibition of the inflammatory phenotype of DCs in colon tissue by MSCs-secreted Gal-3 [58]. In addition to the negative regulation of Th1, galectin-3 inhibits DCs-mediated Th17 polarization in response to the dectin-1 agonist curdlan and lipopolysaccharide (LPS), which acts as an inducer of TLR2/4 pathways and thereby promotes inflammation [111]. Notably, Gal-3^−/−^ DCs produce more prominent levels of the Th17-secreted IL-23 than Gal-3^+ / +^ DCs, highlighting its significant role in MSCs-mediated anti-inflammatory effects in colitis [111]. Likewise, Jo et al. [71] presented that rDCs stimulated by MSCs decreased the severity of colitis in mice by lowering the levels of inflammatory mediators, while favoring IL-10, TGF-β, and Foxp3 levels in lesion sites. The TGFβ and IL-10 accompanied with PGE2 are recognized to serve a key role in the induction of rDCs in colon tissue. Thereby, it seems that MSCs favor rDCs differentiation in a paracrine manner [112]. Irrespective of rDCs, Tregs and responding anti-inflammatory mediators participate in the attenuation of colitis severity following MSCs therapy [113]. For instance, Lu and coworkers [113] showed that systemic administration of gingiva (G)-derived MSCs into a mice colitis model of IBD sustained their OS and relieved disease-associated pathological symptoms. These effects were due to the robust enhancement in Tregs, inhibited release of pro-inflammatory cytokines, and augmented levels of anti-inflammatory cytokines [113]. Importantly, the anti-IL-10R antibody abolished the protective impacts of G-MSCs, thereby indicating the key role of IL-10 in this light [113]. Further, IP administration of UCB-MSCs reduced T cell infiltration into the inflamed colon and, while improving Tregs population in a mouse model of IBD [114]. The treated mice showed lower destruction of the mucosal epithelium and diminished focal crypt lesions and goblet cell loss [114]. These findings offer further proof representative of the significant role of Tregs in protecting against colitis in vivo. Also, systemic injection of AT-MSCs (1 × 10^6^) compromised DSS-induced colitis via two main mechanisms: improving Tregs frequencies and stimulating M2 polarization [115].

Recent study in a TNBS-induced colitis mice model, human UC-MSCs transplantation protected against experimental colitis by promoting CD5 + B cells and IL-10-secreting CD5 + regulatory B cells (Bregs) [116]. It has previously been evinced that deficiency or reduction of Bregs function intensifies intestinal inflammation in mice models and is in association with IBD pathogenesis [117]. In addition to the enhanced Bregs population, human UC-MSCs therapy led to improved Tregs while decreasing Th1/Th17 cell populations in colon tissue of treated mice [116]. Treatment improved OS rate, attenuated symptoms and ameliorated macroscopic and histologic scores in experimental mice [116]. Further analysis demonstrated that MSCs-secreted thrombospondin-1 (THBS1) may boost IL-10 + Bregs and control the development and recurrence of colitis [118]. These results confer the pivotal role of Tregs, rDC, and also Bregs in adjusting IBD progress and clarify its importance for further investigations to provide a new therapeutic avenue.

Currently, up-regulation of endoplasmic reticulum (ER) stress-related proteins was exhibited after systemic infusion of UC-MSCs in an experimental model of colitis [119]. Appropriate ER function, as induced by MSCs therapy, plays a key role in protecting intestinal homeostasis and is required for protein folding, modification, and secretion [120, 121]. The UC-MSCs transplantation reduced the disease activity index (DAI) score, which is composed of the alteration of body weight, diarrhea, hematochezia, and weakened neutrophil infiltration [119]. Also, UC-MSCs injection diminished the destructive effects of MMP2 and MMP9 activation [119]. Thus, down-regulation MMPs can be an efficient therapeutic plan to restrain IBD severity [119]. Reduced levels of MMP-2 are also paralleled by a lessened infiltration of immune cells and pro-inflammatory cytokines levels [119]. Thus, the key function of MMPs and ER stress in the induction of colitis makes them and ideal targets for IBD treatment. Interestingly, MSCs also could sustain IECs proliferation by activating the Wnt signaling pathway and inhibiting apoptosis-inducing proteins [122]. This effect, in turn, leads to amelioration of epithelial integrity and then inhibits uncontrolled signal transduction between the epithelium and adjacent immune cells [123]. Furthermore, MSCs promote the expression of TJs proteins in IECs, thereby reducing inflammation-stimulated permeability [124].

Some proofs imply that combination therapies with MSCs and other therapeutics may ultimately result in more favored effects than alone MSCs therapy in vivo [125–128]. For example, human UCB-derived platelet lysate enhanced the immunomodulatory effects of AT-MSCs in the experimental model of IBD, causing a reduction in colitis scores, growth of the inflamed colon region, and inflammatory cytokine levels [126]. Likewise, IP injection of AT-MSCs in combination with oral administration of sulfasalazine, an anti-rheumatic drug with immunomodulatory potential, could alleviate TNBS-induced colitis in vivo through the improving M2/M1 macrophage ratio, attenuation of monocyte chemoattractant protein-1 (MCP-1/CCL2), CXCL9, and improving IL-10, arginase 1 (Arg-1) levels [127]. The down-regulation of MCP-1 and CXCL9 levels in colon tissue declines the migration and infiltration of monocytes/macrophages, finally lowering colon inflammation [129]. Also, sulfasalazine, in combination with AT-MSCs, was found to downregulate the NF-κB signaling pathway, while enhancing the B-cell lymphoma 2 (Bcl-2)/Bcl-2-associated X protein (Bax) ratio in colon tissue of the rats [127]. The achieved results verify the potent anti-inflammatory and pro-survival capacity of the used combination treatment in treated animals. In another study, wogonin, a natural flavonoid, improved the therapeutic effects of MSCs on DSS-induced colitis in part via increasing IL-10 expression [128]. In vitro analysis showed that wogonin could promote IL-10 secretion from MSCs by prompting transcript factor hypoxia-inducible factor 1-alpha (HIF-1α) expression through up-regulation of AKT/glycogen synthase kinase β (GSK3β) signal pathway [128]. Furthermore, in TNBS-induced rat colitis, granulocyte colony-stimulating factor (G-CSF) addition to MSC improved the recruitment of MSCs to the colonic mucosa and then reduced DAI score, MPO function, TNF-α levels, and NF-κB p65 expression more prominently compared with rats receiving MSCs alone [125].

### Preconditioned MSCs

As described, decreased MSC in vivo activities post-transplantation were supposed to be the chief cause for their restricted therapeutic influences. Thereby, scientists have sought various strategies to augment the therapeutic influences of MSCs. Among them, pre-conditioning has attracted increasing attention. Pre-conditioning mainly depends on a myriad of methods to enhance the MSC's therapeutic competencies in vivo [130]. Hypoxia, incubation with pharmacological/chemical agents or biomolecules (e.g., trophic factors and cytokines), pre-conditioning with physical factors, and finally genetic engineering is the most crucial of them [131]. Such strategies potentiate MSCs’ proliferative, secretory, migratory, and differentiated abilities, providing more favored outcomes in vivo post-transplantation [132, 133]. For instance, [134] found that hypoxic-preconditioned MSCs decrease colon inflammation more evidently compared to normoxic-MSCs largely by up-regulating iNOS expression in MSCs [134]. The miR-216a-5p secreted from hypoxic-MSCs also demonstrates better therapeutic efficiency in experimental colitis in part by promoting the M2 macrophage phenotype [135]. Likewise, AT-MSCs induced with IFN-γ and kynurenic acid substantially upregulated the expression and secretion of IDO-1, finally alleviating CD pathology-like colitis injury and fibrosis in vivo [136]. In addition, MSCs co-cultured with peripheral blood mononuclear cell (PBMC) have demonstrated an upregulated expression of CXCL9 and CXCL10, leading to stronger T cell suppression. PBMC also could increase VEGF, HGF, FGF and CCL2 expression by MSCs [137]. Likewise, MSC were more metabolically active and generated larger amounts of G-CSF, IL-6 and MCP-1 upon exposure with PBMCs [138]. The overview of published studies based on the application of pre-conditioned MSCs is offered in Table 2.

**Table 2:** Preconditioned MSCs transplantation in IBD animal models

Condition	Source	Animal	Results	Refs.
Genetically modified MSCs				
DSS-induced colitis	BM	BALB/c mice	Better homing to the colon and spleen of mice models leads to the reduced Th1 and Th17 cells but improving the Tregs numbers by ICAM-1-overexpressing MSCs	[30]
TNBS-induced colitis	BM	BALB/c mice	Improved homing to the intestinal mucosa and thus causing a more appreciated curative effect by CXCR‑4-overexpressing MSCs	[161]
TNBS-induced colitis	BM	BALB/c mice	More powerful migration to the inflamed colons, causing a strong anti-inflammatory effect by CXCR2-overexpressing MSCs	[162]
DSS-induced colitis	BM	C57BL/6 mice	Down-regulation of miR-141 and miR-139 expression but a promoting ICAM-1 and CXCR4 expression in H19-overexpressing MSCs compared with naïve MSCs	[164]
DSS-induced colitis	Hair follicle	SD rats	Improving intestinal stem cells proliferation (ISC), attenuating inflammatory factors levels, promoting the anti-inflammatory factors, and also reducing DAI score by Nrf-2-overexpressing MSCs	[166]
DSS-induced colitis	DP	SD rats	Transdifferentiation of the HGF-overexpressing MSCs into ISC-like cells and suppressing inflammatory responses as well as oxidative stress	[82]
TNBS-induced colitis	DP	BALB/c mice	Promoting the colon length and supporting intestinal mucosa architecture by enhancing M2 but not M1 macrophage polarization by HIF-1-overexpressing MSCs	[168]
DSS-induced colitis	BM	C57BL/6 mice	Promoting the Tregs and Th2 cells and improving the IDO expression in colon tissue colon by IFN-γ-overexpressing MSCs	[169]
DSS-induced colitis	BM	BALB/c mice	Improving the Tregs population in colon tissue by IL-35 overexpressing MSCs	[170]
DSS-induced colitis	BM	SD rats	Improved homing and robust anti-inflammatory response by CX3CR1/IL-25 dual expressing MSCs	[26]
DSS-induced colitis	UC	CD11b-DTR mice	Suppression of 15-lox-1 expression in macrophage by miR148b-5p-overexpressing MSCs	[245]
TNBS-induced colitis	BM	SD rats	Supporting the colonic morphology and amelioration of tissue structure by KGF-overexpressing MSCs	[246]
Pre-treated MSCs				
DSS-induced colitis	UC	C57BL/6 mice	Amelioration of the clinical signs of disease and reducing colon shortening by poly(I: C)-pre-treated MSCs	[143]
DSS-induced colitis	UC	C57BL/6 mice	Enhancing COX-2, IL-6, and IL-8 expression and targeting macrophage polarization by IL-1β pre-treated MSCs	[145]
DSS-induced colitis	BM	SD rats	Suppression of the Th17 immune response and promoting T regulatory cell phenotype by IL-25-pre-treated MSCs	[148]
DSS-induced colitis	Tonsil	C57BL/6J mice	Potentiating the immunomodulatory potential via up-regulation of the COX-2/PGE2 axis in TNF-ɑ pre-treated MSCs	[139]
TNBS-induced colitis	UC	BALB/c mice	Enhancing the therapeutic competence of MSCs due to the activation of the TLR3–Jagged–1-Notch-1 pathway in poly I: C-pre-treated MSCs	[144]
DSS-induced colitis	BM	C57BL/6 mice	Attenuation of immune cell infiltration and inflammatory cytokines levels in colon tissue by IFN-γ- and poly I: C-pre-treated MSCs	[152]
DSS and DNBS-induced colitis	AT	C57BL/6 mice	Secretion of higher levels of TSG-6 and PGE2 by MSCs upon priming with TNF-ɑ leading to the stimulation of phenotypic alterations in macrophages	[19]

*MSCs* mesenchymal stem/stromal cells, *BM* bone marrow, *UC* umbilical cord, *AT* adipose tissue, *DP* dental pulp, *TNBS* 2,4,6-trinitrobenzene sulfonic acid, *DSS* dextran sulfate sodium, *Th* T helper 1/2/17, *FOXP3* Forkhead box P3, *IL-1* interleukin-1β, *TNF-α* tumor necrosis factor-alpha, *PGE2* prostaglandin E2, *Tregs* regulatory T cells, *IDO* indoleamine 2,3-dioxygenase, *IFN-γ* interferon-gamma, *IL* interleukin, *TLRs* toll-like receptors, *ICAM-1* intercellular adhesion molecule-1, *CXCR-4* C-X-C chemokine receptor type 4, *TSG6* TNFα-stimulated gene-6, *KGF* keratinocyte growth factor, *CX3CR1* CX3C chemokine receptor 1, *MCP-1/CCL2* monocyte chemoattractant protein-1, *HGF* hepatocyte growth factor, *NRF2* nuclear factor erythroid 2-related factor 2, *miRNAs* microRNAs

#### Pre-treated (primed) MSCs

Current consequences have displayed that stimulation with TNF-α boosts PGE2 synthesis in MSCs and thus constrain the proliferation and differentiation of T lymphocytes and macrophages in the inflamed colon in IBD preclinical models [139]. Regardless of the immunomodulatory traits, PGE2 promotes intestinal repair [140], stimulates epithelial cell proliferation through activating the EGFR axis, and inhibits apoptosis by up-regulation of Bcl-2 and NF-κB [141]. Given that the colonic mucosa isolated from IBD patients has lower levels of PGE2 than normal mucosa, it seems that low PGE2 levels may trigger the initiation of inflammation in IBD patients [142]. Also, short in vitro pre-treatment with polyinosinic: polycytidylic acid (usually abbreviated poly I: C) could augment the therapeutic efficacy of UC-MSCs in DSS-induced mice model of colitis [143]. Meanwhile, IP administration of 1 × 10^6^ poly I: C pre-treated UC-MSCs reduced the clinical and histopathological severity of colitis in comparison to the naive UC-MSCs administration [143]. UC-MSCs priming with poly I: C also decreased pro-inflammatory cytokines levels, improved IL-10 levels in colonic tissues, mitigated Th1/17 cell proliferation, and improved Treg differentiation in vivo [144]. Improvement in the production of PGE2 by UC-MSCs in response to TLR3 activation by poly I: C is thought to be responsible for the positive effects observed in treated mice [144]. Besides, although IL-1β is known as the most important inflammatory mediator, IL-1β pre-treated MSCs have shown better efficacy in the treatment of DSS-induced colitis [145]. The pre-treatment of MSCs with IL-1β may modify the balance of immune cells in the spleen and the mesenteric lymph nodes (MLNs) by improving COX-2, IL-6, and IL-8 expression accompanied by augmenting M2/M1 macrophage ratio [145]. Notably, IL-1β also up-regulates CXCR4 expression in MSCs, thereby can improve their migration ability to the inflammatory site of the intestine post-transplantation [145]. Irrespective of colon damage, IL-1β stimulation can improve the homing capacity of MSCs by enriching CXCR4 expression in animal models of liver injuries [146]. Besides, the therapeutic merits of IL-1β pre-treated MSCs as a result of improving their anti-inflammatory influences also have been documented in neurodegenerative diseases [147]. Such effects make IL-1β an efficient biomolecule to sustain MSCs migration and favor their therapeutic efficacy in IBD patients. Likewise, systemic injection of MSCs upon exposure with IL-25 decreased infiltrating inflammatory cells frequencies and enhanced Tregs in serum and colonic mucosa of the IBD rat model, leading to inhibited intestinal inflammation and decreased DAI score [148]. The IL-25 has dual immunomodulatory possessions: it improves Th2-associated immune responses and conversely suppresses Th1 and Th17 cell-associated immune responses [149, 150]. In this light, IL-25 pre-treated MSCs can induce the desired effect on Th1- and Th17-mediated pathological conditions like CD [26]. Further, IL-25 improves the potential of MSC to trigger IECs regeneration, and thus MSC therapy with IL-25 could be a new road for IBD therapy [151]. In addition, Yu and colleagues [80] exhibited that IFN-γ increases the efficacy of human UCB-MSCs transplantation by improving PGE2 release and IDO activity in IBD animal models. PGE2 in association with IDO inhibits Th1 cell differentiation and enhances Tregs differentiation, suggesting that a combination of PGE2 and IDO may be effective therapeutic mediators for potentiating the MSCs-induced immunosuppression [80]. Importantly, IFN-γ pre-treated MSCs may stimulate ISCs proliferation and enterocyte differentiation in vivo, thus easing intestinal repair in IBD murine models [152]. As well, TNFα and IFNγ treatment caused rapid consumption of glucose and metabolic skewing toward glycolysis in MSCs, largely increasing the efficacy of MSCs in IBD [153]. Molecular analysis signified that PI3K–AKT signaling axis was rapidly induced and required for the skewing toward glycolysis stimulated by TNFα and IFNγ [153].

Based on the recent finding, hypoxic pre-conditioning can promote the anti-inflammatory and proliferative potential of MSCs in colitis in part by promoting NO production through iNOS activity in MSCs [134]. The NO adjusts various key activities of the GI mucosa, including maintenance of adequate perfusion, adjustment of microvascular and epithelial permeability, and controlling of the immune response [154]. In the inflamed regions, MSCs-secreted NO alleviates oxidative stress [155] and suppresses NF-κB translocation, thereby exerting a protective effect on intestinal cells [156]. Albeit, aberrant expression of NO have a pathogenic role in UC [157], but not CD [158], highlighting the importance of conducting more comprehensive studies on the various aspect of MSCs-secreted NO on IBD progress or therapy.

#### Genetically modified MSCs

The therapeutic potential of MSCs has been assessed in various reports, particularly concerning their immunomodulatory and pro-regenerative traits. Notwithstanding, limited engraftment and inadequate favorable influences of MSCs illuminates the necessity of designing novel strategies to improve their survival, migration, and beneficial capability. Genetic engineering of MCSs has been developed as a valued tool to trigger the expression of diverse proteins and soluble mediators with an extensive spectrum of utilities like microRNAs, growth factors, enzymes, cytokines, chemokines, and transcription factors [159, 160].

Current reports exhibit that overexpression of various genes such as intercellular adhesion molecule (ICAM) [30], CXCR4 [161], and CXCR2 [162] can improve MSCs homing to the injured area and thus enhance succeeding anti-inflammatory and pro-survival effects in IBD animal models. Meanwhile, Liu et al. displayed that ICAM-1-overexpressing MSCs improved recovery and diminished pathological damages in the IBD mice model more evidently than naïve MSCs [30]. ICAM-1 overexpressing MSCs also reduced Th1 and Th17 subpopulation while enhancing Tregs frequency in the spleen of treated mice, ensuring down-regulated IFN-γ and IL-17A and upregulated Foxp3 levels [30]. Likewise, CXCR‑4 overexpressing MSCs induced better migration and homing potential in IBD murine model and also exerted superior influences on treating colitis compared with naïve MSCs [161]. Also, they enhanced the levels of occludin and VEGF in treated IBD murine, suggesting their capacity to alleviate colitis by supporting intestinal integrity and mucosa repair [161]. As described, a reduction in pivotal TJs proteins like occludin and ZO-1 is observed in both IBD and experimental models of inflammation [163]. Studies in UC patients exhibit that attenuation of occludin expression and enhancement in claudin 1/occludin ratio has a close association with UC severity [163]. Thus, MSCs injection can diminish disease severity in IBD patients by improving the expression of ZO-1 and occludin and consequently repairing the intestinal barrier [29]. In addition, overexpression of long non-coding RNA H19 (H19) may decrease miR-139 and miR-141 expression in MSCs, thereby promoting the functions of their responding targets ICAM-1 and CXCR4, respectively [164]. Mechanistically, upregulation of ICAM-1 and CXCR4 in H19-overexpressing MSCs potentiates their migration and homing post-transplantation [164]. H19 also can trigger IECs proliferation and precede the expression of TJ-related proteins, thus heightening intestinal integrity [165].

Other studies have focused on enriching the anti-oxidant potential of MSCs using genetic engineering. Nuclear factor erythroid 2-related factor (Nrf2) signaling adjusts multiple gene expressions by making interfaces with the anti-oxidant response element (ARE). Up-regulation of the Nrf2/ARE axis dampens numerous pathologic mechanisms correlating with the autoimmune response and also IBD. Recently, Zhou and colleagues [166] showed that systemic administration of Nrf-2-overexpressing hair follicle (HF)-MSCs (1 × 10^6^ cells) had a more prominent therapeutic effect in colitic rats compared with naïve MSCs. Nrf-2-overexpressing HF-MSCs favored intestinal integrity, improved IL-13 and IL-10 expression in colon tissue, and reduced DAI scores in treated rats [166]. Genetically modified HF-MSCs to overexpress Nrf-2 [166] and HGF [82] also could attenuate oxidative stress in colitic rats, as documented by a decline in malondialdehyde (MDA) and myeloperoxidase (MPO), while promoting SOD. Genetically modified MSCs to overexpress HGF also could decrease TNF-α and IFN-γ levels, enhance IL-10 levels, upregulate the expression of TJ-related protein ZO-1, and finally promote the proliferation of IECs in radiation-induced intestinal injury (RIII) [167]. Thereby, promoting HGF expression may be an efficient approach to ameliorate IBD as a result of down-regulation of inflammation and maintaining intestinal integrity.

Genetic modification of MSCs to overexpress HIF-1 [168], IFN-y [169], and IL-35 [170] exhibited great potential to raise MSCs’ anti-inflammatory and pro-regeneration capabilities. Accordingly, HIF-1 overexpressing MSCs could stimulate M2 macrophage polarization in a TNBS-induced mouse colitis model and also improve colon length and intestinal mucosa integrity [168]. Besides, IFN-γ overexpressing MSCs could induce more strong immunosuppressive impacts on the proliferation of T cells than naive MSCs in vitro [169]. The systemic injection of manipulated MSCs to overexpress IFN-γ, in turn, attenuated the severity of colitis, as evidenced by improved body weight, enhanced colon length, reduced DAI score, and amelioration of small intestine tissues structure [169]. The positive effects largely were dependent on the promoted Tregs and Th2 cells frequencies both in mesenteric lymph node and spleen, increased IDO expression accompanied by decreased inflammatory cytokine levels in colon tissue of treated models [169]. Further, IL-35 has currently been described as an immunosuppressive cytokine acting as an inhibitor of chronic inflammatory and autoimmune diseases [171, 172]. Wang et al. [173] exhibited that IL-35 recombinant protein has multiple anti-inflammatory impacts in IBD experimental models, including reducing the infiltrations of macrophages, CD4 + T, and CD8 + T cells and potentiating the infiltration of Treg cells. In this light, Yan et al. [170] evaluated the potent effects of IL-35 overexpressing MSCs in a dextran sodium sulfate (DSS)-induced colitis mice model. IL-35 overexpressing MSCs displayed superiority over naïve MSCs in terms of colon length and Tregs population in colon tissue of treated mice. They concluded that engineered MSCs to overexpress IL-35 could mitigate IBD severity largely via lowering the levels of the pro-inflammatory cytokine [170].

### MSCs-derived exosome

#### Exosome superiority over parental MSCs

Exosomes are a most eminent subtype of extracellular vesicles (EVs) with a diameter in the variety of 30–100 nm. They are secreted by human cells, such as stem cells, and target biological processes in recipient cells [174]. Exosomes include various biomolecules like proteins, lipids, messenger RNA (mRNA), and microRNAs (miRNAs) as cargo. Their contents also could be varied depending on the stem cell environment. They are produced and released in a highly adjusted process: construction of endocytic vesicles by invagination of the plasma membrane, formation of multivesicular bodies (MVBs) following endosomal membranes' inward budding, and lastly, integration of MVBs with the plasma membrane and secretion of the vesicular contents called exosome [175]. Intestine tissue repair following MSCs treatment is predominantly ensured by MSCs-induced paracrine effects, reflecting the prominence of MSCs-derived exosome application rather than parental MSCs therapy. Certainly, MSCs-derived exosomes influence multiple biological processes in target cells as same as parental MSCs, while attenuating concerns regarding the direct application of parental cells, such as cell aging and potential tumor formation [176]. They exhibit better stability in circulation, biocompatibility, low immunogenicity and toxicity, and also strong targeting ability than parental cells [177], hence delivering a more favored therapeutic effect. However, more preclinical and clinical design investigations are needed to compare the efficacy of stem cells and exosomes.

#### Application of MSCs-derived exosome in IBD

Like MSCs, the positive effects of MSCs-derived exosome therapy in IBD conditions rely on two main mechanisms: inhibition of inflammation and supporting intestinal barrier [178, 179]. Exosome therapy could mitigate weight loss and colon shortening by activating the anti-inflammatory reactions and hindrance of the inflammatory axis in vivo [178]. As well, MSC-derived exosome (200 μg) offer a better outcome than parental MSCs transplantation at a dose of 1 × 10^6^ cells. These studies indicate the anti-inflammatory impacts of exosomes in IBD animal models [178]. Further, other studies signified that MSCs-derived exosomes could reduce the IBD severity in animal models by up-regulation of IL-10 while attenuating TNF-α, IL-1β, IL-6, iNOS, and, more importantly, IL-7 levels in colon tissues and spleens of treated animals [180–182]. Alteration in the cytokine expression profiles upon exosome treatment potentiates M2 macrophage polarization and also improves Tregs differentiation [183]. IL-7 acts as a master regulator of T-cell differentiation and stimulates the expression of cell adhesion molecules (CAMs) and MCP-1, leading to increased immune cell infiltration into colon tissue [184]. As a result, targeting IL-7 expression may bring about the impaired migration and infiltration of immune cells to colon tissue, thus promoting anti-inflammatory effects. Also, OE-MSCs-derived exosomes inhibited the differentiation of Th1/17 cells but supported Treg cells differentiation in IBD experimental models by improving IL-10 and TGF-β levels [73]. Because of the positive effects of the IL-10 and TGF-β on intestinal hemostasis, improvement of their levels by MSCs treatment could alleviate IBD symptoms [185]. Of course, overexpression of TGF-β in the colons of mice may trigger colonic fibrosis [186]. Thus, a controlled level of TGF-β is required for tissue hemostasis. Current reports also note that miR-125a and miR-125b enriched exosomes downregulate Th17 cell differentiation through inhibition of STAT3 expression, which is required for early Th17 cell development [187]. Respecting to previous reports, the reduced miR-125a and miR-125b levels and conversely improved STAT3 levels have a positive association with disease severity in IBD patients [188], and thus exosome treatment may cause positive outcomes in IBD patients. Apart from its contribution to the Th17 development, the roles of SAT3 in the regulation of IEC's fate during colitis have been evinced [189].

Administration of the exosomes carrying miR-378a-5p also resulted in down-regulation of NLRP3 inflammasomes, impairment of cell pyroptosis, and thus increased cell survival in DSS-induced colitis in mice [190]. Likewise, BM-MSCs-derived exosomal miR-539-5p could inhibit pyroptosis by NLRP3/caspase-1 signaling to bypass IBD progression in mice models [191]. In addition, Xu et al. [192] suggested that MSCs-derived exosome could ameliorate colitis through the suppression of casp11/4-induced macrophage pyroptosis. They found that exosome carrying miR-203a-3p.2 could suppress casp4-induced macrophage pyroptosis in an inflammatory environment. As described, NLRP3 participates in the pathogenesis of IBD by substantial activation of inflammation in IECs and continued activation of macrophages [190]. As a result, targeting its expression and activation by therapeutic modalities such as MSCs treatment [78, 193] or NLRP3-specific inflammasome inhibitors [194, 195] offer an anti-inflammatory milieu and finally protect against IBD. Molecular analysis also has exhibited that exosomal miR-181a can affect the intestinal microbiota, immune responses, and intestinal barrier integrity in colitic rodents [108]. As a diminished level of miR-181a was found to be involved in the enhanced susceptibility to IBD development [196], improvement of its level in the inflamed colon by therapeutic modalities (e.g., exosome treatment) may give rise to better therapeutic outcomes. Meanwhile, Gu et al. [108] showed that miR-181a containing MSCs-derived exosome improved claudin-1, ZO-1, and NF-κB inhibitor (IκB) levels in colon tissue of exosome-treated mice, in addition to the reduction of pro-inflammatory cytokine levels. Indeed, although reduced levels of miR-181a and the TJs proteins such as ZO-1 and occludin are detected in IBD patients [197], exosome treatment improved their expression and thus ameliorated intestinal integrity and impaired inflammatory response. Also, metallothionein-2 (MT-2) in exosomes seems to be required for the suppression of inflammatory responses, enabling IBD treatment in preclinical models [198]. MTs, a negative regulator of NF-kB, adjust the inflammation and homeostasis of heavy metals and ameliorate oxidative stress [199]. Interestingly, MT^−/−^ mice show severe inflammatory responses which induce tissue damage [200]. Liu et al. found that systemic injection of MT-2-containing MSCs-derived exosome improved survival and stool consistency, reduced rectal bleeding and colon shortening, increased M2 macrophage activity, and attenuated MPO activity, ensuing colon tissue repair in colitic mice [198].

Irrespective of the suggested mechanisms, exosomes can attenuate DSS-induced colitis in mice by controlling of ubiquitin modification level [28]. Ubiquitination serves a significant role in the adjustment of multiple biological activities (e.g., regulation of inflammation) [201]. Increasing evidence showed that E3 ubiquitin ligases like ring finger protein (RNF) 183, RNF 20, A20, Pellino 3, tripartite motif-containing 62 (TRIM62), and Itch contribute to the development of IBD [202]. For instance, RNF183 up-regulates the NF-κB pathway by promoting the ubiquitination and subsequent degradation of IκBα [202]. Recent reports revealed that exosome therapy profoundly augmented the proliferating ability of colon mucosa epithelial cells and concomitantly reduced the expression of ubiquitin and its related molecules, NEDD8 activating enzyme E1 (NAe1), ubiquitin-conjugating enzyme E2M (UBE2M), and ubiquitin-like modifier activating enzyme 3 (Uba3), in the colon tissues and spleens of DSS-induced IBD mice [28]. These results confer that targeting ubiquitination can make possible the down-regulation of expression of pro-inflammatory cytokine and chemokine and thus may be a valued target to modify the inflammatory axis in IBD. In addition to ubiquitination, neddylation, a recently described post-translational modification, contributes to the development of IBD mediated by potentiating DCs maturation [203, 204]. However, down-regulation of the neddylation attenuates mucosal inflammation by reducing cytokine production, inhibiting the expression of the costimulatory molecules, and thereby hindrance of T cell stimulation [204]. Accordingly, a recent study showed that miR-326 carrying MSCs-derived exosome might enable inhibition of the neddylation process and thereby relieves DSS-induced IBD [205]. Recent reports also have clarified that MSC-derived exosome could improve the gut microbiota composition by substantially supporting the structure of OTUs and colitis-induced reduction in α-diversity, enhancing the frequency of 'healthy' bacteria, attenuating disease-associated bacteria and detrimental functions, and promoting other vital cellular functions [206]. In addition, MSCs-derived exosome could convey miR-378a-3p to downregulate the GATA-binding protein 2 (GATA2) expression, which downregulates aquaporin-4 (AQP4) to block the peroxisome proliferator-activated receptor α (PPAR-α) signaling pathway, finally inhibiting the incidence of IBD [207]. Further, Zhang et al. (2022) demonstrated that exosome can improve intestinal lymphatic drainage, suppress lymphangiogenesis, and macrophages infiltration by the miR-302d-3p/VEGFR3/AKT axis to alleviate IBD [208]. On the other hand, hypoxic MSCs-derived exosomes reduced UC injury by restricting IES reactive oxygen species accumulation and DNA damage mainly by upregulation of HIF-1α expression and function [209].

A summary of studies based on the therapeutic merits of MSCs-derived exosomes is provided in Table 3.

**Table 3:** MSCs-derived EVs (e.g., exosome) transplantation in IBD animal models

Diseases	Source	Model	Results	Refs.
DSS-induced colitis	BM	C57BL/6 mice	Alleviation of colitis by up-regulation of miR-125a and miR-125b levels, which in turn, downregulates STAT3 expression and Th17 cell differentiation	[187]
DSS-induced colitis	UC	KM mice	Enhancing IL-10 levels while reducing the TNF-α, IL-1β, IL-6, iNOS, and IL-7 levels in colon tissues and spleens of treated mice	[180]
DSS-induced colitis	AT	C57BL/6 mice	Reducing the colon shortening, enhancing body weight, attenuating bleeding and colon injury by promoting the IFN-γ, TNF-α, IL-12, and IL-17 levels and conversely decreasing TGF-β, IL-4, and IL-10 in lymph node and spleen of treated mice	[183]
DSS-induced colitis	UC	C57BL/6 mice	Reducing DAI score and body weight loss by exerting anti-inflammatory responses and averting inflammatory responses	[178]
DSS-induced colitis	BM	C57BL/6 mice	Alleviation of experimental colitis by enhancing the intestinal barrier function through exosomal miR-181a	[108]
DSS-induced colitis	DP	C57BL/6 mice	Restoring the Th17 cell/Treg balance by activating the miR-1246/Nfat5 axis leading to less disease severity	[247]
DSS-induced colitis	DP	C57BL/6 mice	Suppression of CD4 + T cell proliferation along with down-regulation of IL-17, IFN-γ levels and improving the TGF-β, IL-10 secreted by T cells	[73]
DSS-induced colitis	UC	BALB/c mice	Attenuation of colitis by inhibition of ubiquitin activity	[28]
DSS-induced colitis	DP	C57BL/6 mice	Inhibition of inflammatory responses by exosomal metallothionein-2 (MT-2)	[198]
DSS/TNBS-induced colitis	UC	BALB/c mice	Supporting the mucosal barrier repair and intestinal immune homeostasis by exosomal TSG-6	[29]
DSS-induced colitis	BM	C57BL/6 mice	Exerting epithelial regeneration	[248]
DSS-induced colitis	BM	C57BL/6 mice	Promoting intestinal-stem-cell (ISCs) and epithelial regeneration	[249]
DSS-induced colitis	UC	BALB/c mice	Alleviation of colitis in part by inhibiting neddylation through exosomal miR-326	[205]
DSS -induced colitis	UC	BALB/c mice	Attenuation of colitis by suppression of macrophage pyroptosis through activating miR-378a-5p/NLRP3 axis	[190]
DSS -induced colitis	BM	BALB/c mice	Attenuation of body weight loss, DAI score, and colon mucosa damage probably by improvement in IL-10 and TGF-β reduction in VEGF-A, IFN-γ, IL-12, TNF-α, CCL-24, and CCL-17 levels	[250]
TNBS-induced colitis	UC	BALB/c mice	Suppression of inflammation and oxidative stress	[23]
DSS -induced colitis	AT	SD rats	Diminishment of the number of circulating inflammatory cells by combined melatonin and exosome therapy	[251]

*MSCs* mesenchymal stem/stromal cells, *EVs* extracellular vesicles, *BM* bone marrow, *UC* umbilical cord, *AT* adipose tissue, *DP* dental pulp, *TNBS* 2,4,6-trinitrobenzene sulfonic acid, *DSS* dextran sulfate sodium, *Th* T helper 1/2/17, *FOXP3* Forkhead box P3, *IL-1* interleukin-1β, *TNF-α* tumor necrosis factor-alpha, *Tregs* regulatory T cells, *IFN-γ* interferon-gamma, *IL* interleukin, *TLRs* toll-like receptors, *miRNAs* MICRORNAS, *TGF-β* transforming growth factor-beta, *VEGF* vascular endothelial growth factor, *NFAT* nuclear factor of activated T-cells, *NLRP3* NLR family pyrin domain containing 3, *CCL* chemokine-ligand 17/24, *NFAT* nuclear factor of activated T-cells

## Clinical trials

The promising results from animal studies have encouraged researchers to design and conduct a variety of clinical trials. Meanwhile, both autologous and allogeneic MSCs transplantation has been accomplished given their immune-suppressive and regenerative competencies with remarkable safety and acceptable efficacy [210]. With respect to published reports, both systemic and local delivery could engender reasonable success in IBD patients [211–213].

### Autologous

Recent trials in 5 patients with refractory Crohn’s fistulas exhibited that intracolonic administration of autologous AT-MSCs (35 × 10^6^ cells/patient) had no stern side effects while causing the complete cessation of drainage in 3 of them during six weeks of follow-up [211]. Another phase 1 trial in 12 patients with CD (NCT03803917) exhibited that AT-MSCs intracolonic administration was safe and enabled complete fistula healing in 57% of patients [214]. The major adverse effect was postprocedure proctalgia enduring a few days. Also, two patients experienced small abscesses, 1 had urinary retention, and 1 had minor bleeding during liposuction [214]. Likewise, the safety and feasibility of AT-MSCs injection (1–2 × 10^7^ cells/patient) was demonstrated in a phase 1 trial in CD patients [215]. As well, autologous BM-MSCs (1–2 × 10^6^ cells/kg) by intravenous route did not provoke serious side effects in 10 patients with refractory Crohn's fistulas [216]. Fortunately, the intervention reduced DAI during 6 weeks post-treatment in 3 of them. In contrast, 3 of them required surgery because of disease worsening [216]. Further, Dhere et al. [217] reports evidenced modest safety and feasibility of systemic injection of autologous BM-MSCs (1 × 10^7^ cells/kg) in 12 patients with CD.

### Allogeneic

A trial in 82 patients (41 patients in either control or intervention group) displayed that systemic injection of allogeneic 1 × 10^6^ UC-MSCs/kg can attenuate DAI, Harvey–Bradshaw index (HBI), and corticosteroid dosage without serious side effects [218]. Moreover, local administration of allogeneic 3 × 10^7^ MSCs/patients improved the healing of perianal fistulas [219]. Also, intracolonic injection of 3–9 × 10^7^ allogeneic BM-MSCs/patient resulted in smaller fistula tracts after 4 years with no long-term side effects in CD patients [220]. In a phase I/IIa clinical trial, local administration of allogeneic AT-MSCs (20 × 10^6^ cells/patient) into 24 patients with CD caused a decrease in the number of draining fistulas (69.2% of the patients), complete closure of the treated fistula (56.3% of the patients) and complete closure of all existing fistula tracts (30% of patients) [221]. Achieved results implied the safety, feasibility, and also efficacy of allogeneic AT-MSCs administration in CD patients [221]. In addition, Forbes and coworkers [222] accomplished a phase 2 trial (NCT01090817) and displayed that allogeneic MSCs transplantation (2 × 10^6^ cells/kg weekly for 4 weeks) decreased DAI and CD endoscopic index of severity (CDEIS) scores in patients with luminal CD refractory to biologic therapy. Interestingly, a phase 3 randomized, double-blind controlled trial was conducted at 49 hospitals in eight countries from July 6, 2012, to July 27, 2015, to evaluate the safety and efficacy of local administration of allogeneic, expanded, AT-MSCs (Cx601) as a capable novel therapeutic tool to treat IBD [223]. Intralesional injection of 120 × 10^6^ Cx601 cells/patients in 107 CD patients showed an acceptable safety profile and significant efficacy in CD patients who did not respond to conventional or biological treatments [223]. Recently, MSCs therapy was well tolerated and results exhibited that clinical remission post-treatment with MSCs may be sustained for up to 104 weeks in patients with perianal fistulizing CD [224].

## Conclusion and future direction

**Fig. 3:**
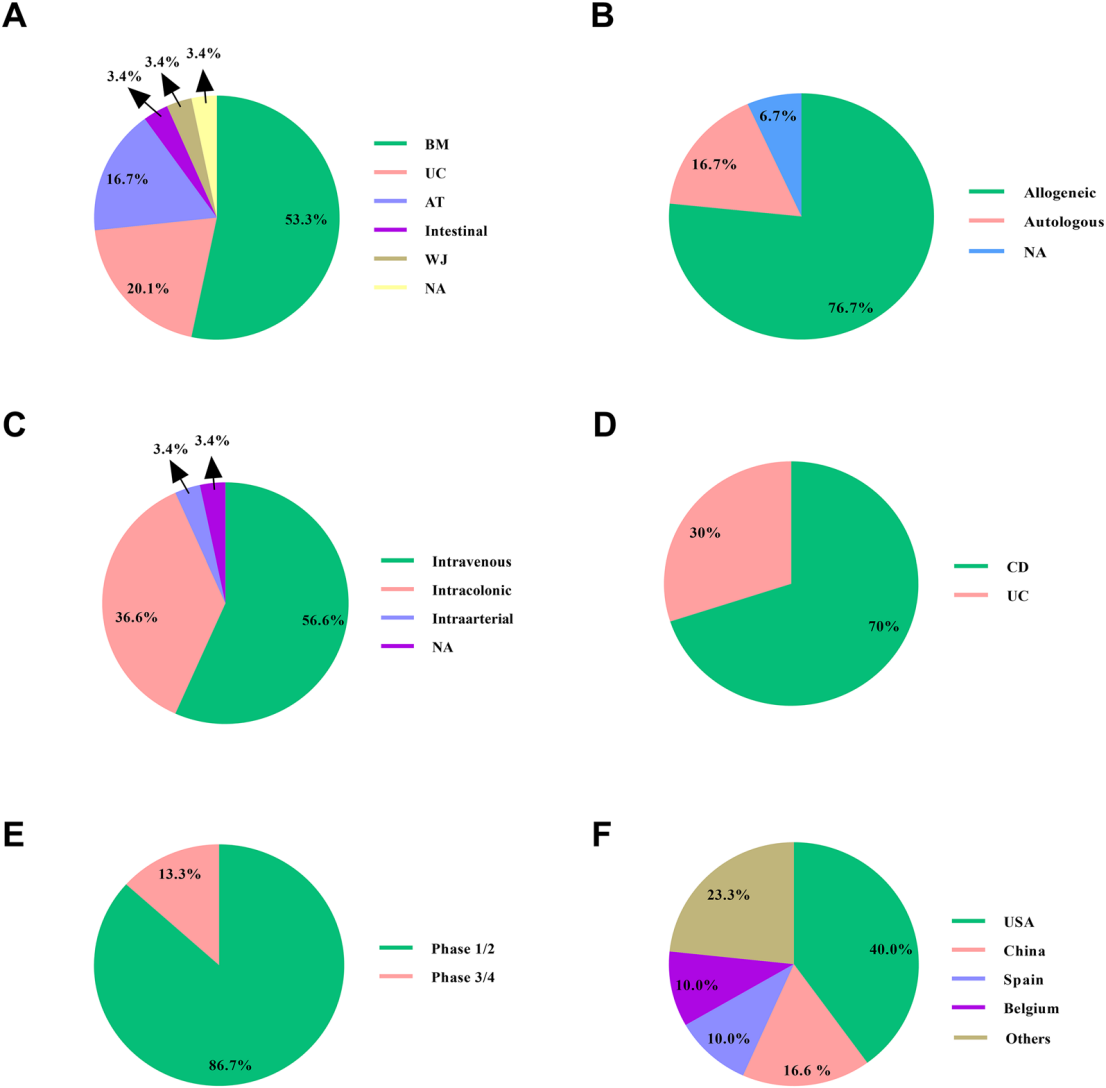
Clinical trials based on MSCs therapy in IBD conditions registered on https://clinicaltrials.gov (June 2022). The schematic exhibits the clinical studies in the light of cell source (**A**), cell type (**B**), administration route (**C**), condition (**D**), study phase (**E**), and study location (**F**). Inflammatory bowel diseases (IBD), mesenchymal stem/stromal cell (MSC), bone marrow (BM), umbilical cord (UC), adipose tissue (AT), Wharton's jelly (WJ), Crohn's disease (CD), ulcerative colitis (UC), not applicable (NA)

MSC therapy has attracted increasing attention in the context of IBD therapy, given its low immunogenicity along with pro-survival and anti-inflammatory competencies. These influential inherent possessions have ensured the success of MSCs treatment in IBD animal models. A myriad of clinical studies also has investigated the safety and efficacy of MSCs therapy in IBD patients (Table 4, Fig. 3). Among various cell sources, BM-MSCs have been the most widely used cells, followed by UC- and AT-derived MSCs. Among cell administration routes, intravenous as well as intracolonic routes have been the most common routes. In most studies, the dose injected was about 1–2 × 10^6^ cells/kg. Although the short-term safety and feasibility of MSCs transplantation have been verified, some drawbacks must be circumvented to use in the clinic. In this light, long-term side effect of MSC therapy hurdle their application. Also, the poor migration and engraftment of transplanted MSCs, particularly when injected by the intravenous route, is another potential fence. Hence, designing novel strategies to promote their engraftments, such as cell priming using safe ingredients or genetic modification of MSCs, is suggested. Further, the MSC investigations must also note the patient selection, disease activity, and disease stage based on therapeutic efficacy. In addition, as MSCs-derived exosomes show better engraftment, exosome therapy has been proposed as an alternative treatment for parental MSCs. The main downside of exosome therapy is low yield, which hurdles its clinical application. Importantly, this drawback can be chiefly tackled by substituting the traditional 2D culture system with a 3D system. For instance, Kim et al. have found that 3D spheroid culture of BM-MSCs brings about more exosomes than 2D culture and also the non-adherent round cell morphology itself may be a causative factor [225]. These results offer dependable data to evolve an optimal process for the mass generation of exosomes. In addition, hollow fiber 3D culture system may offer prolonged manufacture of MSC-exosome with better immunoregulatory competencies in vivo [226]. In sum, we suggest that pre-conditioned MSCs-derived exosomes can be a more appropriate therapeutic option compared with other modalities to attenuate disease severity in IBD patients.

**Table 4:** Clinical trials based on MSCs transplantation in IBD patients

Disease	Phase	Source	Administration route (dose)	Participant Number	Study location	Status	NCT number
CD	1	Allogeneic UC	Intravenous	15	Antigua and Barbuda	Recruiting	NCT05003947
UC	1/2	Allogeneic WJ	Intravenous	20	Jordan	Unknown	NCT03299413
CD	2	Allogeneic BM	Intravenous	10	USA	Completed	NCT00294112
CD	1	Allogeneic BM	NA	10	Iran	Unknown	NCT01874015
CD	1/2	Autologous AT	Intracolonic	15	Spain	Completed	NCT01157650
UC	1/2	Allogeneic AT	Intracolonic	8	Spain	Unknown	NCT01914887
CD	1/2	Allogeneic UC	Intravenous	82	China	Completed	NCT02445547
CD	1/2	Allogeneic BM	Intravenous	13	Belgium	Terminated	NCT01540292
CD	1	Allogeneic BM-EVs	Intravenous	10	USA	Not yet recruiting	NCT05130983
UC	1/2	Allogeneic UC	Intravenous	50	China	Unknown	NCT01221428
CD	1/2	Allogeneic BM	Intracolonic	21	Netherlands	Completed	NCT01144962
CD	1/2	Allogeneic BM	Intracolonic	40	USA	Recruiting	NCT04519684
CD	1/2	Allogeneic BM	Intracolonic	40	USA	Recruiting	NCT04519697
UC	1/2	Allogeneic BM	Intracolonic	20	USA	Not yet recruiting	NCT05075811
CD	1	Allogeneic UC	Intracolonic	7	Malaysia	Not yet recruiting	NCT05039411
CD	1	Allogeneic BM	Intracolonic	10	USA	Recruiting	NCT04791878
CD	3	Allogeneic AT	Intracolonic	278	International	Completed	NCT01541579
CD	1/2	Allogeneic BM	Intracolonic	24	USA	Recruiting	NCT04548583
UC	1/2	Allogeneic AT	Intracolonic	50	China	Recruiting	NCT03609905
CD	3	Allogeneic BM	Intravenous	330	International	Completed	NCT00482092
UC	1/2	Allogeneic UC	Intravenous	30	China	Unknown	NCT02442037
CD	3	Allogeneic BM	Intravenous	98	International	Completed	NCT00543374
UC	1/2	Allogeneic BM	Intracolonic	24	USA	Recruiting	NCT04543994
CD	1	Allogeneic UC	Intracolonic	24	China	Enrolling by invitation	NCT04939337
UC	1	Autologous AT	Intraarterial	20	USA	Recruiting	NCT04312113

*MSCs* mesenchymal stem/stromal cells, *EVs* extracellular vesicles, *BM* bone marrow, *UC* umbilical cord, *AT* adipose tissue, *WJ* Wharton's jelly, *CD* Crohn's disease, *UC* ulcerative colitis, *NA* not applicable

## Data Availability

Not applicable.

## Abbreviation

MSCs: Mesenchymal stem/stromal cells
AT: Adipose tissue
BM: Bone marrow
UC: Umbilical cord
miRNAs: MicroRNAs
TNFα: Tumor necrosis factor α
TGF-β: Transforming growth factor-beta
TSG6: TNFα-stimulated gene-6
IECs: Intestinal epithelial cells
PGE2: Prostaglandin E2
Tregs: Regulatory T cells
Th: T helper
IL: Interleukin
TJs: Tight junction
TNBS: 2,4,6-Trinitrobenzene sulfonic acid
DSS: Dextran sulfate sodium
