# Editorial Expression of Concern: Therapeutic potential of mesenchymal stem/stromal cells (MSCs)-based cell therapy for inflammatory bowel diseases (IBD) therapy

**DOI:** 10.1186/s40001-024-02140-8

**Published:** 2024-11-15

**Authors:** Mohamed J. Saadh, Maria V. Mikhailova, Soheil Rasoolzadegan, Mojgan Falaki, Roozbeh Akhavanfar, José Luis Arias Gonzáles, Amir Rigi, Bahman Abedi Kiasari

**Affiliations:** 1 Department of Basic Sciences, Faculty of Pharmacy, Middle East University, Amman, 11831 Jordan; 2 I.M. Sechenov First Moscow State Medical University (Sechenov University), Moscow, Russia; 3 Department of Surgery, School of Medicine, Shahid Beheshti University of Medical Sciences, Tehran, Iran; 4 Department of Internal Medicine, Shahid Beheshti University of Medical Sciences, Tehran, Iran; 5 School of Medicine, Isfahan University of Medical Sciences, Isfahan, Iran; 6 Pontifcia Universidad Católica del Perú, Lima, Peru; 7 Department of Nursing, Young Researchers and Elite Club, Zahedan Branch, Azad University, Zahedan, Iran; 8 Virology Department, Faculty of Veterinary Medicine, The University of Tehran, Tehran, Iran

**Editorial Expression of Concern: European Journal of Medical Research (2023) 28:47** 10.1186/s40001-023-01008-7

The Editors-in-Chief are issuing an Editorial Expression of Concern to alert readers that this article underwent unusual changes to the authorship list during the submission process. The authors were unable to provide a satisfactory explanation for these changes when asked for clarification by the Publisher. Readers are advised to interpret the contributions of the authors to this article with caution.

The authors did not respond to correspondence from the Publisher regarding the publication of this Editorial Expression of Concern.

